# Cardiac Rehabilitation and Cardiovascular Prevention in Patients with Type 2 Diabetes Mellitus: From Initial Assessment to Comprehensive Management

**DOI:** 10.3390/jcm14217791

**Published:** 2025-11-03

**Authors:** Cristina Andreea Adam, Joanna Popiolek-Kalisz, Buket Akinci, Giovanna Manzi, Irfan Ullah, Eirini Beneki, Florin Mitu, Ladislav Batalik, Marina Ostojic, Francesco Perone

**Affiliations:** 1 Department of Medical Specialties I, “Grigore T. Popa” University of Medicine and Pharmacy Iasi, University Street No. 16, 700115 Iasi, Romania; adam.cristina93@gmail.com (C.A.A.); mitu.florin@yahoo.com (F.M.); 2 Department of Clinical Dietetics, Medical University of Lublin, ul. Chodzki 7, 20-093 Lublin, Poland; popiolekkalisz@gmail.com; 3 Department of Cardiology, Cardinal Wyszynski Hospital in Lublin, al. Krasnicka 100, 20-718 Lublin, Poland; 4 Department of Physiotherapy and Rehabilitation, Faculty of Health Sciences, Biruni University, 34015 Istanbul, Turkey; barbuket@hotmail.com; 5 Biruni University Research Center (B@MER), Biruni University, 34015 Istanbul, Turkey; 6 Department of Clinical, Internal, Anesthesiology and Cardiovascular Sciences, Policlinico Universitario Umberto I, Sapienza University of Rome, 00161 Rome, Italy; giovannamanzi91@gmail.com; 7 University Hospitals Cleveland Medical Center, Cleveland, OH 44106, USA; irfanullahecp2@gmail.com; 8 Department of Cardiology, Lausanne University Hospital and University of Lausanne, 1005 Lausanne, Switzerland; e.beneki@hotmail.com; 9 Academy of Medical Sciences, 030167 Bucharest, Romania; 10 Academy of Romanian Scientists, 700050 Iasi, Romania; 11 Department of Physiotherapy and Rehabilitation, Faculty of Medicine, Masaryk University, 62500 Brno, Czech Republic; batalik.ladislav@fnbrno.cz; 12 Department of Rehabilitation, University Hospital Brno, 62500 Brno, Czech Republic; 13 Department of Public Health, Faculty of Medicine, Masaryk University, 62500 Brno, Czech Republic; 14 Department of Rehabilitation, Faculty of Medicine, Masaryk University, 62500 Brno, Czech Republic; 15 Cardiology Clinic, University Clinical Center of Serbia, 11000 Belgrade, Serbia; drmarinaostojic@gmail.com; 16 Cardiac Rehabilitation Unit, Rehabilitation Clinic ‘Villa delle Magnolie’, 81020 Castel Morrone, Caserta, Italy

**Keywords:** diabetes mellitus, cardiovascular prevention, cardiac rehabilitation, cardiovascular risk, aerobic exercise training, resistance exercise training

## Abstract

Diabetes mellitus increases the risk of developing coronary artery disease, stroke, aortic disease, heart failure, atrial fibrillation, and peripheral arterial disease. This condition negatively impacts prognosis by increasing the risk of future cardiovascular (CV) events. Patients with type 2 diabetes mellitus need a comprehensive and personalized assessment and definition of the CV risk profile. In very high-risk individuals, special attention is required due to the high risk of adverse events despite appropriate management and treatment. Key interventions to reduce this risk include CV prevention and cardiac rehabilitation. Traditional and non-traditional CV risk factor management, dietary modifications, regular physical activity, aerobic and resistance exercise training, psychosocial and frailty management, optimal pharmacological therapy, and investigation of comorbidities are recommended to reduce the development of CV disease and mortality. Therefore, our manuscript provides updated and critical evidence on the comprehensive management of patients with type 2 diabetes mellitus in clinical practice from the perspective of CV prevention and cardiac rehabilitation, with a focus on individuals at very high risk. Further, practical guidance on individualizing exercise prescriptions based on patient-specific risk profiles and comorbid conditions is provided.

## 1. Introduction

In recent years, the increasing complexity of patients due to the aging population and the rising burden of comorbidities has made the work of cardiologists extremely difficult. A proactive and multifactorial approach is pivotal to best treat patients of the 21st century [1]: knowledge and skills traditionally belonging to other specialists are required for the good modern cardiologist.

One of the best examples concerns the management of type 2 diabetes mellitus (T2DM) that, as nicely reported in the editorial by Ozkan and Ndumele, is more than sugar [2]. It impairs quality of life, may cause disability and confers a 2-fold greater risk for cardiovascular diseases (CVD) compared to non-diabetic counterparts, with a significant economic and social burden [3]. It is estimated that more than 75% of diabetic patients aged over 40 will eventually die from CVD [4]. Recent epidemiological data underline the increasing global prevalence of T2DM, expected to reach over 700 million patients by 2045. In this scenario, clinicians must do their best to stop this worrying tsunami by increasing patients’ awareness of their disease, encouraging them to improve their lifestyle and choosing the best individualized pharmacological therapy. Nowadays, cardiologists should be familiar with drugs originally developed to lower blood glucose in patients with T2DM, such as sodium-glucose co-transporter-2 (SGLT2) inhibitors, given their beneficial effects on CV outcomes across various populations.

**Figure 1:**
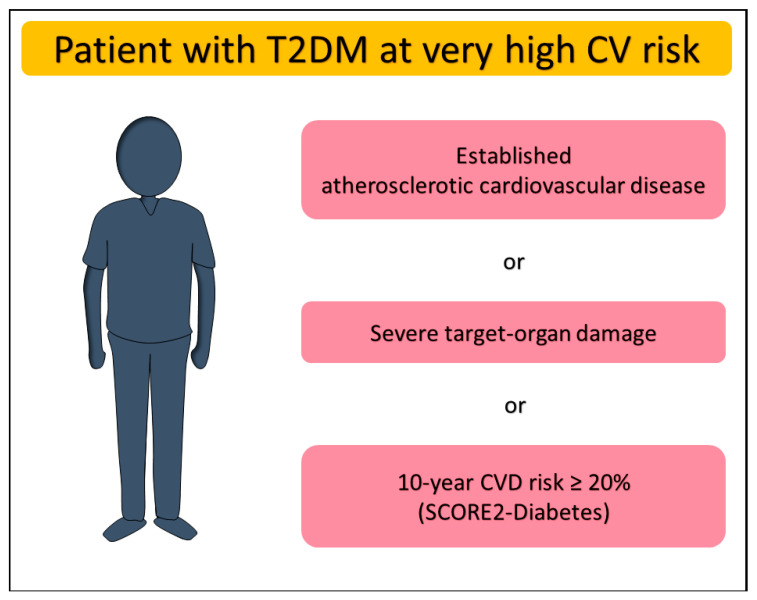
Definition of very high cardiovascular risk in patients with type 2 diabetes mellitus. CV, cardiovascular; CVD, cardiovascular disease; T2DM, type 2 diabetes mellitus.

Simple risk assessment scores support physicians in decision making for T2DM as well as other heart diseases. In patients with T2DM aged over 40 years, without atherosclerotic cardiovascular disease (ASCVD) or severe target-organ damage (TOD), the 2023 European Society of Cardiology (ESC) guidelines [5] recommend estimating the 10-year risk of fatal or non-fatal CVD events through the SCORE2-Diabetes (class and level of recommendation: IB). Derived from a total of 229.460 participants with diabetes and no history of CVD at baseline, the model has then been recalibrated and externally validated in individuals with T2DM across four European countries, ultimately showing greater discrimination power than SCORE2 [6]. The new model integrates general data (e.g., age, sex, systolic blood pressure, total and high-density lipoprotein cholesterol) with diabetes-related information such as glycated hemoglobin (HbA1c) value, age at diabetes diagnosis and markers of kidney function. The SCORE2-Diabetes indicates very high risk in DM patients with a 10-year CVD risk ≥ 20%. Furthermore, patients with T2DM are classified as being at very high CVD risk if they have already established ASCVD or severe TOD such as renal impairment defined as estimated glomerular filtration rate (eGFR) < 45 mL/min/1.73 m^2^ irrespective of albuminuria, or eGFR of 45–59 mL/min/1.73 m^2^ with microalbuminuria (urinary albumin-to-creatinine ratio [UACR] 30–300 mg/g), or proteinuria (UACR > 300 mg/g), or microvascular disease affecting at least three sites (e.g., microalbuminuria, retinopathy, and neuropathy) (Figure 1) [5].

Therefore, the aim of our manuscript is to provide healthcare professionals, particularly physicians, cardiologists, diabetologists, and rehabilitation specialists, with practical guidance for the management of this cohort of patients, addressing different areas ranging from CV prevention to cardiac rehabilitation (CR), to personalized pharmacological treatment.

## 2. Cardiovascular Prevention and Risk Factor Management in Very High T2DM Patients

CVD prevention is crucial to dramatically improve the prognosis in T2DM patients. A multifactorial and multidisciplinary approach is suggested to manage these individuals towards a healthy lifestyle [5]. Furthermore, DM is frequently accompanied by other risk factors, such as hypertension and dyslipidemia, and these uncontrolled comorbidities additionally increase CVD risk. Lifestyle modification is the foundation of preventive actions to reduce CVD risk, especially in individuals at very high risk (Table 1). This includes dietary modifications, weight reduction, physical activity, and smoking cessation [7]. Strategies to improve adherence to lifestyle modifications include patient-centered education, motivational interviewing, digital health tools (mobile apps, wearables), and structured telemedicine follow-up.

Dietary modifications should combine recommendations for CVD prevention and DM management. Dietary patterns should limit saturated fatty acids to <10% of total energy intake, replacing them with poly- and monounsaturated fatty acids [8]. Unsalted nuts are a good source of these healthy fats and should be consumed in amounts of 30 g per day [9]. Practical examples include daily consumption of a handful of almonds, walnuts, or hazelnuts. Contrary, trans unsaturated fatty acids consumption should be minimized, as their intake is strongly associated with increased CVD risk [10]. Avoiding processed foods, particularly processed meat, effectively reduces trans fatty acids and sodium intake. It is helpful, as sodium consumption should be limited to <5 g/day [11]. Patients should be educated on how to recognize and reduce hidden sodium intake (e.g., limiting intake of bread, cheese, and sauces). Moreover, red meat should also be reduced to 350–500 g/week and it can be replaced by fatty fish (recommended 1–2 servings per week). These changes overall promote adopting more plant-based food patterns, which are beneficial in terms of preventing DM, improving glycemic control in patients with DM, and overall, reducing CVD risk. The beneficial effect of a plant-based diet on the risk of developing T2DM has been demonstrated by several meta-analyses published in the last year in the scientific literature. The beneficial effect exceeds the risk of developing T2DM or metabolic syndrome by up to 50%, with some studies even showing a 29% reduction in the risk of cerebrovascular disease and a 40% reduction in the risk of coronary events [12].

Such research conducted by Qian et al. [13] demonstrated that high adherence to this type of diet is inversely proportional to the risk of T2DM (relative risk 0.77), while in the case of lower adherence, the results are heterogeneous. Among the main protective mechanisms implicated is the high content of vitamins, minerals, and antioxidants, as well as the consumption of foods such as yogurt and nuts, which, although animal-based, have proven to have a protective role in cardiovascular health [14,15].

Furthermore, Satija et al. [16] developed a plant-based diet index based on the analysis of approximately 200,000 questionnaires providing semi-quantitative information on dietary habits revealed by the frequency of consumption of certain foods. The researchers assigned a positive score to plant-based foods and a negative score to animal-based foods, demonstrating that a positive score was associated with a lower risk of coronary heart disease. However, the results regarding the long-term protective effect on cerebrovascular disease are controversial. Among patients with high adherence to a plant-based diet, an analysis of a cohort of approximately 700,000 patients showed a reduction in cardiovascular risk and coronary heart disease, but not in stroke (relative risk 0.87) [17]. A recent meta-analysis last year highlighted the beneficial role of this diet on glycemic control, with a vegetarian diet being associated with improved glycated hemoglobin (−0.39%, *p* = 0.001), but without showing a statistically significant reduction in fasting blood glucose (−0.36 mmol/L, *p* = 0.301) [18].

Carbohydrate intake should be based on low-glycemic index foods, such as whole grains, which also provide fiber [19]. Daily fiber intake should range from 30–45 g, and it can be derived from fruits, vegetables, and whole grains [20]. That is why, fruits and vegetables should be consumed in servings of ≥2–3 per day (≥200 g/day each). Practical meal examples include vegetable-based soups, salads with legumes, and whole-grain pasta dishes with vegetables. Diabetic patients should also be educated to compose meals with a low glycemic load to avoid glycemic fluctuations [21]. Moreover, vegetables and fruits provide essential vitamins and antioxidants too, which may potentially impact low-grade inflammation associated with metabolic syndrome. What is more, sugar-sweetened beverages, such as soft drinks and fruit juices, should be avoided due to their adverse effects on CVD risk and glycemic control. The WHO recommends limiting free sugar intake (mono- and disaccharides) to <10% of total energy. Alcohol consumption should be limited to <100 g/week, as recent studies show no protective effects of moderate alcohol consumption versus abstinence for ASCVD prevention [22]. Any alcohol consumption can uniformly increase blood pressure and body mass index (BMI) and affect hepatic gluconeogenesis, leading to potential glycemic fluctuations. Patients should be educated on practical strategies to reduce alcohol intake, including alcohol-free days and replacing alcoholic beverages with alternatives (e.g., water, herbal teas, or sugar-free beverages).

All these dietary recommendations align with the Mediterranean diet, which is associated with a reduced CVD risk and proven long-term efficacy in weight management [23]. The DASH (Dietary Approaches to Stop Hypertension) diet, originally designed for blood pressure reduction, is very similar to the Mediterranean diet and is also recommended for patients [24]. For overweight or obese patients, creating a caloric deficit leads to significant weight loss and better glycemic control [25]. Physical activity is helpful to achieving an energy deficit and preventing muscle mass loss. Specifically, practical physical activity recommendations include at least 150 min/week of moderate aerobic exercise (e.g., brisk walking, cycling, swimming) and resistance training (RT) at least twice per week [9].

Instead, regarding risk factor management, pharmacotherapy with ACE inhibitors (ACEI) and calcium channel blockers (CCB) or diuretics is first-line treatment for patients with hypertension. The ESC Guidelines for the management of elevated blood pressure and hypertension indicate targets of 120–129/70–79 mmHg for DM patients [26] (class I, level A recommendation according to the ESC guidelines [5]). Individualization based on patient tolerance and risk of adverse effects is critical, especially in older or frail patients. In dyslipidemia management, the low-density lipoprotein-cholesterol (LDL-C) target should be <55 mg/dL and reduced by at least 50% in these patients at very high risk, achieved through a two-step approach (class I, level B recommendation according to the ESC guidelines [5]). The initial step targets LDL-C levels of 70 mg/dL or lower, progressing to the final goal. First-line pharmacotherapy includes statins and ezetimibe, if the target LDL-C is not achieved with statins alone. If these targets are not met, proprotein convertase subtilisin/kexin type 9 (PCSK9) inhibitors should be considered [5,27]. Emerging therapies, such as inclisiran or bempedoic acid, may also be considered, especially in patients intolerant to statins [9].

DM itself is a major CVD risk factor, making glycemic control a crucial aspect of CVD prevention. The target HbA1c level should be <7.0% (class I, level A recommendation according to the ESC guidelines [5]), achievable with the introduction of SGLT2 inhibitors or GLP-1 receptor agonists [5]. In patients with coexisting heart failure (HF), SGLT2 inhibitors are also indicated as part of HF pharmacotherapy [28,29]. Moreover, combined therapy with SGLT2 inhibitors and GLP-1 receptor agonists should be considered due to their complementary cardiovascular and metabolic benefits [5]. New pharmacological agents such as SGLT2 inhibitors or GLP-1 receptor agonists, used in the treatment of T2DM, have a beneficial effect on carbohydrate metabolism, implicitly modulating the functional status of patients included in cardiovascular recovery programs (class I, level A recommendation according to the ESC guidelines [5]). The pleiotropic effects of iSGLT2 are well known, and numerous studies in the literature recommend their administration in light of the cardiovascular and renal benefits associated with patients who also have HF, regardless of their glycemic status [30]. Furthermore, cardiovascular rehabilitation programs have an integrative effect, associated with both physical training programs and psychotherapy, which increases adherence and quality of life, or hypoglycemia therapy, which, by modulating complex biohumoral pathophysiological mechanisms, contributes to the reversal of LV remodeling, while also having a long-term antiarrhythmic impact [31]. The use of GLP-1 agonists in patients enrolled in a cardiovascular recovery program should be individualized according to the duration and severity of T2DM, having an adjuvant effect in terms of improving endothelial dysfunction, reducing pro-inflammatory status, or improving the lipid profile. Numerous studies confirm the beneficial role of this class of drugs in reducing MACE, while also noting the need to monitor potential adverse effects, which are predominantly digestive (nausea, vomiting) [32,33,34].

In addition to classical risk factors, other factors may exacerbate the CVD risk and should be addressed, especially in very high-risk patients. Psychosocial stress is dose-dependently associated with ASCVD development and progression [35]. Therefore, screening for depression, anxiety, and insomnia is recommended, with adequate support implemented as needed. Practical interventions include psychological counseling, cognitive behavioral therapy, mindfulness-based stress reduction, or digital mental health interventions (e.g., mobile apps). The first clinical consensus published by the ESC on mental illness highlights the importance of managing patients with CVD (including risk factors such as T2DM) and mental disorders within a multidisciplinary team—the Psycho-Cardio team—while also emphasizing the need to raise awareness and education levels among the population and to adopt a stepped care approach [36]. In addition, some drugs used to treat psychiatric disorders have metabolic effects, thereby increasing CV risk [37]. Antipsychotics (especially second-generation ones) such as olanzapine, clozapine, or quetiapine have side effects such as weight gain, hypertriglyceridemia, and T2DM, with some patients developing metabolic syndrome. Zotepine and clozapine are associated with the highest risk of hyperglycemia [38,39].

Another interesting link is that between DM and frailty, identified by ESC guidelines as a potential modifier of overall CVD risk [40]. It is a multidimensional condition independent of age and multimorbidity, increasing vulnerability to stressors. ESC guidelines identify frailty as a potential modifier of global CVD risk. Screening for frailty is essential and should be addressed through non-pharmacological approaches, such as nutritional interventions, micronutrient supplementation, exercise training, and social engagement. Frailty should also be considered when individualizing pharmacological and device-based treatments. Practical tools such as frailty screening questionnaires (e.g., Clinical Frailty Scale) are recommended.

Environmental factors also influence CVD risk. Air pollution, along with other media, such as soil and water pollution, are associated with CVD risk modification [41]. Patients at very high CVD risk, including those with DM, may benefit from minimizing long-term exposure to high-pollution regions. Practical recommendations include patient education on minimizing exposure to pollution, such as indoor air purifiers and planning physical activities outside peak pollution hours.

## 3. Cardiac Rehabilitation in T2DM Patients

Patients with DM have a 2–4 times higher risk of developing CVD. Insulin resistance, chronic inflammation, oxidative stress, hypercoagulability, elevated blood pressure, or dyslipidemia are common pathophysiological elements that justify CR (cardiac rehabilitation) programs in this category of patients, especially in those at very high risk [42]. The common profile in CR is the patient with very high-risk DM due to clinically established ASCVD or severe TOD. Furthermore, T2DM is the main comorbidity affecting patients referred to the CR [43]. Given the complexity of these patients, it is crucial to adopt an individualized approach within a multidisciplinary team involving cardiologists, diabetologists, rehabilitation physicians, physiotherapists, dietitians, and psychologists.

In these individuals, the initial assessment before starting the CR program should include evaluation of cardiovascular risk profile and risk factors, glycaemic control, symptoms and physical examination, investigation of comorbidities, electrocardiogram, biological tests (including fasting blood glucose and HbA1c), echocardiography, physical activity level, and frailty assessment. Furthermore, before starting the program, a cardiopulmonary exercise testing (CPET) is recommended to assess peak exercise capacity and silent myocardial ischemia [44]. Investigation of comorbidities and diabetes end-organ assessment should always be performed to evaluate microvascular, macrovascular and psychosocial status [45]. Indeed, peripheral retinopathy has a high risk of hemorrhage or retinal detachment which is why it is recommended to avoid exercises that increase blood pressure or involve Valsalva maneuvers (e.g., lifting weights). Autonomic neuropathy could cause a reduced cardiac response to physical exercise. Diabetic peripheral neuropathy (DPN) frequently causes peripheral ulcers and the appearance of the diabetic foot (Charcot morphology), being recommended in these patients to avoid physical training based on weight lifting.

**Figure 2:**
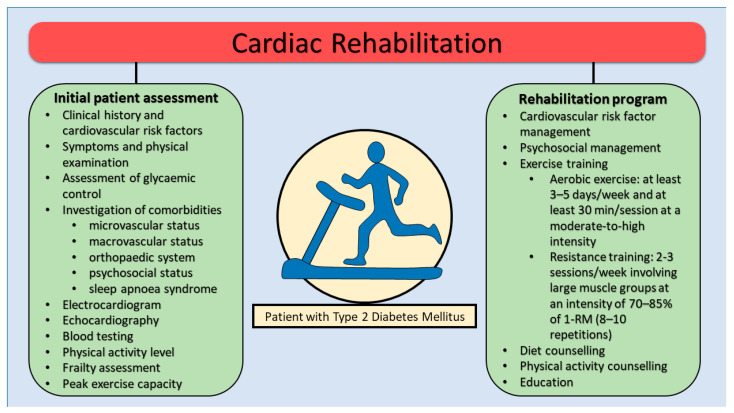
Cardiac rehabilitation program in patients with type 2 diabetes mellitus. RM, repetition maximum.

CR programs for diabetic patients are based on a multidisciplinary approach, which combines the beneficial effects of physical training with diet, correction of modifiable cardiovascular risk factors, psychological counseling, smoking cessation, and weight management (Figure 2). Additionally, telemedicine interventions and digital health technologies (e.g., wearable activity trackers, mobile apps for self-monitoring) have demonstrated potential in enhancing adherence and effectiveness of CR programs in diabetic populations [46,47]. These programs also have a series of additional objectives represented by obtaining optimal glycemic control, adequate nutrition, maintaining an appropriate activity level along with optimal pharmacological therapy. Finally, special attention should be paid to infection prevention, foot care or early detection of mechanical complications with implications on the efficiency of the CR program [44].

Exercise prescription is based on the FITT principle (frequency, intensity, type, time). Physical activity is an essential component of the CR program in diabetic patients, contributing to the improvement of glycemic control and body composition along with cardiorespiratory fitness, correction of the glucose and lipid profile, as well as weight and blood pressure reduction (Table 2). The optimal duration of a CR program in T2DM patients should be at least 12 weeks, extending up to 6 months depending on clinical response and patient characteristics.

An important aspect in this category of patients is the observance of certain precautionary measures related to carbohydrate management, which have a role in preventing acute events such as hypoglycemia or hyperglycemia [48]. The risk of adverse events correlates with the intensity of physical training; in the case of low and moderate intensity, the risk is low. In insulin therapy, the most common adverse event is hypoglycemia. Exercise of short duration and high intensity may lead to hyperglycemia or ketoaemia in response to strenuous exercise. In patients with DM, glycemic control is recommended before exercise and rehabilitation sessions should be contraindicated in case of a recent history of unstable glycaemic control and blood glucose level < 90 mg/dl not reversed by carbohydrate intake within 30 min. Glycemic control is recommended before exercise and during and after physical activity to avoid hypoglycemia [48].

Aerobics and RT are suggested in T2DM patients during rehabilitation programs. Specifically, an aerobic exercise program of at least 3–5 days/week and at least 30 min/session at a moderate-to-high intensity is recommended [42,44]. In the case of diabetic patients who are sedentary, it is recommended to start low-intensity exercise initially to increase adherence as well as decrease the risk of injury or adverse events. The type of training is dictated by the associated complications, with cycling at 60–70% of maximum heart rate or swimming being recommended over walking in obese patients or those with microvascular involvement (especially neuropathy) [49,50,51,52]. On the other hand, for patients with T2DM who are at very high risk, a structured R program should be carefully designed to maximize benefits while minimizing risks. RT is recommended for patients with T2DM, with or without CVD, at least 2–3 times per week in addition to aerobic training. RT should involve large muscle groups, with an intensity of 70–85% of the individual’s one repetition maximum (1-RM), aiming for 8–10 repetitions per set. Gradually increase the volume to 2–4 sets per muscle group, reaching at least 21 sets per week [5,42,44]. It is recommended that high-volume RT, when combined with aerobic exercise training, has been shown to provide significant benefits for glycaemic control, body composition, and muscle strength in patients with or without CVD [26].

Meta-regression analyses revealed that the number of repetitions per set was a significant predictor of RT’s efficacy on HbA1c. Subgroup analyses indicated that the most pronounced reductions in HbA1c and FBG occurred with a training duration of 12–16 weeks, intensities of 70–80% of 1-RM, training frequencies of 2–3 times per week, 3 sets per session, 8–10 repetitions per set, and less than a 60 s rest interval [53]. Further research is needed to determine the ideal RT dosage for very high-risk patients with T2DM [44]. Studies showed a strength gain with RT superior to aerobic exercise training, but comparable improvements in glycaemic control, body composition, muscle mass and cardiorespiratory fitness in patients with T2DM [42,54,55]. Exercise programs lead to a 0.6% reduction in HbA1c levels in individuals with T2DM, with the greatest benefits observed when endurance and RT are combined [5,56]. Moreover, improvements in insulin sensitivity, which are associated with improved peripheral and coronary endothelial function as well as coronary blood flow, can be achieved with combined aerobic and RT [57]. On the other hand, an increasing number of studies have highlighted the unique benefits of RT in the optimization of glycaemic control and controlling risk factors. While aerobic exercise—whether performed alone or alongside RT—can significantly enhance glycaemic control, it often necessitates prolonged activity sessions, which may be difficult or even uncomfortable for certain groups, such as those who are overweight, obese, or suffering from knee or hip osteoarthritis. Jansson et al. found that RT led to a significant reduction in HbA1c levels compared to the control group (weighted mean difference = −0.39, 95% CI −0.60 to −0.18). The training effect influenced the outcomes, with greater gains in muscular strength associated with more substantial decreases in HbA1c (β = −0.99, 95% CI −1.97 to −0.01). A secondary analysis of this study indicated that there were no significant differences in HbA1c levels between RT and aerobic training [58]. While RT has been shown to enhance VO_2_ peak, its impact is significantly lower than that of aerobic exercise training in patients with T2DM. A combined approach integrating aerobic and RT is more effective, as it combines the oxidative benefits of aerobic exercise with the improvements in muscle mass and function from RT [42].

T2DM is independently associated with an accelerated decline in muscular strength and, partly because of hyperglycemia, may lead to reduced joint mobility [59]. Combining aerobic endurance and RT, particularly at higher intensities, influences muscle anabolism by affecting AMPK/PCG-1α and mTOR activation. However, more studies are needed to assess the long-term impact of RT on muscular fitness and its role in reducing cardiovascular risk factors including lipid profile and blood pressure in patients with T2DM [60,61,62]. Furthermore, there is a dose–response relationship between intensity and volume of exercise and duration of glucose uptake by skeletal muscle that may last up to 48 h after exercise. These factors must be considered in patients with T2DM who are undertaking intensive exercise or competitive sports to avoid hypoglycaemia [59].

The RT program should begin with multi-joint exercises targeting major muscle groups before incorporating single-joint exercises. To promote balanced muscle development and reduce fatigue, exercises should be alternated between upper and lower limbs and between agonist and antagonist muscle groups. The recommended exercise speed is slow to moderate, with a concentric phase lasting approximately 2 s and an eccentric phase of 2 to 4 s, ensuring each set lasts at least 40 s. Training volume and intensity should be progressively increased based on individual response. RT should be performed at a moderate, controlled speed through a full range of motion, avoiding a constant tight grip. Training must stop immediately if symptoms like vertigo, arrhythmia, shortness of breath, or chest pain occur [63]. Patients with T2DM, particularly with microvascular complications such as neuropathy and retinopathy should avoid the Valsalva maneuver during RT to prevent significant blood pressure fluctuations. For T2DM patients with coexisting hypertension, performing fewer repetitions at higher intensity during dynamic RT is recommended to maximize strength gains while minimizing blood pressure variability [64]. It was also emphasized that an exercise regimen designed to reduce blood pressure should primarily consist of aerobic exercise training in these patients [5,57]. For frail or older T2DM patients, lower intensity RT (20–30% 1-RM) with 10–15 repetitions are recommended, particularly during the initiation phase of 3–6 weeks [5]. The ideal duration for RT should be at least 3–6 months for these patients to obtain muscle enhancement. Combined training should also be supplemented by flexibility and balance exercises, particularly in older T2DM patients or patients with DPN [59].

Inspiratory muscle training (IMT) has been demonstrated to improve respiratory muscle function and optimize ventilatory responses during exercise [65,66,67]. Consequently, IMT may offer significant benefits for patients with T2DM, especially in cases of reduced inspiratory muscle strength (PImax < 70% of predicted value). The recommended initial IMT intensity is set at 30% of PI max, with a gradual increase up to a maximum of 60%. Adjustments in intensity should be made approximately every 7–10 days, utilizing either threshold or resistive loading devices. Training sessions should last between 20 and 30 min per day and be conducted 3–5 times per week for a minimum of eight weeks. Incorporating IMT alongside aerobic exercise or a combination of aerobic and RT is also advised [44].

Walking is recommended as the first-line training modality for patients with T2DM and lower extremity artery disease, with RT considered when walking is not feasible [5,68]. RT has been shown to significantly improve walking performance, including claudication onset distance and total walking distance in treadmill and 6 min walk tests in patients with peripheral arterial disease (PAD) [69,70]. Depending on program intensity, RT may be as effective as supervised walking exercises with higher-intensity RT yielding greater improvements in walking ability [69,70]. Functional capacity benefits are observed in both short-term (12-week) and longer-term (24-week) RT, with longer durations producing larger effects [71]. Furthermore, RT has been shown to improve the ankle-brachial index in individuals with DPN [72]. These findings highlight the value of incorporating RT into treatment programs for patients with T2DM and concurrent PAD. On the other hand, lower-limb exercise training including RT is not recommended in patients with chronic limb-threatening ischaemia and contraindicated until ulcers are healed, and aggressive offloading should be ensured to allow healing [68].

A combination of aerobic and RT restores small sensory nerve damage, reduces symptoms, and improves muscle function in patients with DPN [73]. Since DPN patients often experience deconditioning and mobility issues, RT is suggested as a more suitable option than aerobic training and may help improve their ability to engage in aerobic exercises later [73]. RT induces marked improvement of muscle performance, neuromuscular remodeling and may alleviate neuropathic pain in patients with DPN [74]. The reduction in DPN progression due to RT may result from its ability to improve factors like hyperglycemia, dyslipidaemia, insulin resistance, systemic inflammation, and neuro-hormonal growth factor deficiency [75]. High-load RT (75–80% of one-repetition maximum) performed once a week for as little as 16 weeks with multiple sets (3 sets of 12 repetitions) can significantly improve muscle performance [76]. However, for highly deconditioned DPN patients, low to moderate-intensity RT (30–60% of 1-RM) is a safer and more tolerable option. This approach, involving single (1 set of 10–20 repetitions) or multiple sets (2–3 sets of 8–12 repetitions) targeting 5–10 muscle groups, should be prioritized [73]. Incorporating a variety of exercises, such as balance training, core stability exercises, Tai Chi, and proprioceptive training can play a pivotal role in enhancing balance and posture in individuals with DPN [77]. These interventions help address sensory and motor deficits associated with the condition, ultimately reducing the risk of falls and improving overall functional mobility and quality of life. Further research is needed to better understand the adaptability of sensory and motor nerve damage to RT and other exercise-based rehabilitation methods in DPN.

The feet of patients with T2DM, especially those with DPN or PAD, should undergo thorough examination prior to initiating an exercise program and at least annually thereafter. Individuals with active foot lesions or ulcers should avoid weight-bearing RT exercises and prioritize non-weight-bearing alternatives. Additionally, it is strongly recommended that patients with T2DM use properly fitted, high-quality athletic footwear to reduce the risk of injury and enhance foot protection [57].

In addition, psychological support, including structured counseling, stress-management techniques, mindfulness-based interventions, and education about disease management and lifestyle modifications should be routinely integrated into CR programs. Psychosocial support enhances patient adherence and facilitates sustained lifestyle changes, crucial for long-term outcomes [78].

Telemedicine and digital health technologies (e.g., smartphone applications, wearables, virtual CR sessions) can substantially enhance patient adherence, engagement, and long-term outcomes. These tools allow clinicians to remotely monitor exercise adherence, physical activity, and glycemic control, providing real-time feedback and personalized support [79].

Patients with T2DM at very high risk often require specialized and trained personnel to manage these patients, mainly due to the potential complications that may arise during physical training. While the data in the literature show a low level of referral to specialized centers for CV patients, in the case of patients with T2DM, the challenges and barriers associated with implementation are more pronounced. (Table 3) [80,81,82].

## 4. Tailored and Comprehensive Management of Very High-Risk T2DM Patients in Clinical Practice

Patients with T2DM at very high risk require personalized and comprehensive management in daily clinical practice due to the high risk of cardiovascular events as well as cardiovascular and all-cause mortality. Furthermore, patients with T2DM who follow a CR program require a patient-centered approach that includes risk stratification, setting individual blood glucose targets, administering medication with proven protective effects (iSGLT2, GLP-1 receptor agonists, metformin), modulation of cardio-metabolic risk, prevention & frequent screening of potential complications, and frequent monitoring (Table 4).

**Figure 3:**
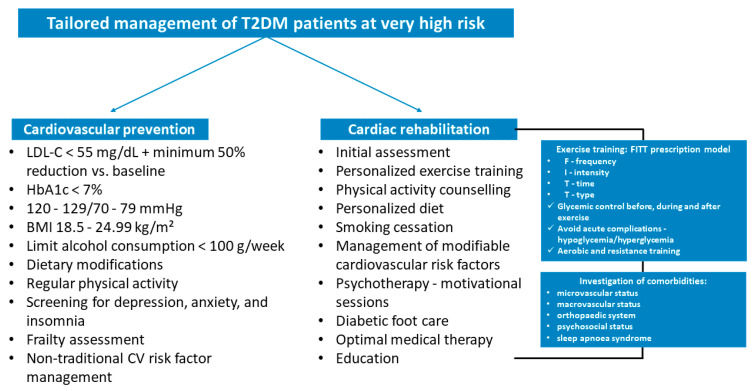
Tailored management of type 2 diabetes mellitus patients at very high risk in clinical practice. BMI, body mass index; CV, cardiovascular; FITT, frequency, intensity, time, and type; HbA1c, glycated hemoglobin; LDL-C, low-density lipoprotein cholesterol; T2DM; type 2 diabetes mellitus.

Cardiovascular prevention and CR are crucial to improve prognosis and reduce future adverse events. These two interventions should be suggested during the management of these very high-risk patients, individualizing the strategies based on individual patient characteristics (Figure 3). In addition to the prescription of personalized physical training, the correction of modifiable cardiovascular risk factors is imperative. In this type of patients at very high risk, it is recommended to reach a LDL-C target of less than 55 mg/dL and reduce a baseline LDL-C below 50%. The three main lipid-lowering agents suggested are statins, ezetimibe, and PCSK9 inhibitors [5]; however, newer cholesterol-lowering drugs, such as inclisiran and bempedoic acid, may be considered [66].

Hypertension is present in a significant percentage of diabetic patients and, in these individuals, it is recommended to maintain systolic blood pressure below 130 mmHg, if tolerated, but not lower than 120 mmHg with lifestyle modification as well as antihypertensive medication in selected cases. HbA1c target is defined according to comorbidities, diabetes duration, and life expectancy. It is recommended to reach a HbA1c < 7%. The use of SGLT2 inhibitors and/or glucagon-like peptide 1 receptor agonists (class I indication) in very high-risk diabetic patients is suggested with the aim of reducing long-term cardiovascular risk [5,83].

In individuals with overweight or obesity, BMI between 18.5–24.9 kg/m^2^ is recommended by reducing of weight and increasing physical exercise. Patients should receive structured education on weight management, complemented by practical dietary advice and structured exercise planning. Glucose-lowering agents up to bariatric surgery should be considered to reach this goal. Regular physical activity is strongly suggested to improve the prognosis, cardiovascular risk profile, and metabolic control. It is suggested to perform 150 min/week of moderate exercise intensity or 75 min/week of vigorous endurance intensity [5].

Dietary modifications, limiting alcohol consumption < 100 g/week, psychosocial and frailty assessment, and non-traditional cardiovascular risk factor management complete the prevention strategies needed to protect these patients from adverse events. In addition, CR is suggested in Class I in T2DM at very high risk. This intervention is fundamental to improve cardiorespiratory fitness, metabolic control, quality of life, and prognosis. Compliance with dietary recommendations, participation in psychotherapy sessions, smoking cessation, exercise training, cardiovascular risk factor management, and weight loss are key elements of the CR program in these patients that require a multifactorial strategy for implementation. Last but not least, telemedicine offers easy-to-use tools for patients, which can increase adherence to the proposed changes, and provide remote monitoring tools [84,85].

A major problem among patients with type 2 DM at very high risk is adherence to the recommendations suggested, both for lifestyle changes and medication. Recent epidemiologic data point out that achieving optimal control of risk factors associated with DM reduces the risk of CVD by more than 50%, but less than 20% of patients achieve the targets recommended by clinical practice guidelines [86].

Finally, a specific assessment of comorbidities is advised before starting a training program to manage macrovascular and microvascular complications (nephropathy, retinopathy, peripheral or autonomic neuropathy) and psychosocial status. Exercises that raise blood pressure or require Valsalva movements (such as lifting weights) should be avoided in patients with peripheral retinopathy due to the increased risk of hemorrhage or retinal detachment. The presence of autonomic neuropathy defined by resting tachycardia, orthostatic hypotension, decreased cardiac variability, cardiorespiratory instability, and silent myocardial infarction is associated with high cardiovascular risk in diabetic patients. It is advised that individuals with peripheral neuropathy refrain from engaging in weightlifting-based physical exercise since these patients often develop peripheral ulcers and the formation of diabetic foot (Charcot morphology).

The presence of peripheral neuropathy requires additional precautions in the care of the diabetic foot in order to prevent complications, such as ulcers, vascular disease, reducing the risk of amputation. Early detection of local changes in the context of a regular exercise program may require decreasing their intensity, changing the type of exercise performed or even stopping exercise for a period of time. An extensive peripheral vascular assessment of the lower extremities is recommended in all diabetic patients prior to enrollment in a CR program [45,87]. Finally, practical management algorithms tailored to different patient scenarios, including consideration of patient preferences, comorbidities, and socioeconomic factors, should be clearly defined and utilized to enhance the effectiveness of preventive and rehabilitative interventions.

## 5. Digital Health Impact on CV Programs in High-Risk T2DM—Opportunities & Barriers

The concept of digital health and the increasingly widespread applications of AI in cardiology and particularly in CR. The degree of implementation in medical practice varies depending on the level of development of the medical system, with difficulties identified in several areas, associated with patients, doctors and medical systems alike [88]. Marios et al. [89] conducted a study that included a number of 39 patients with T2DM divided into 2 groups: a tele-monitored group versus control in a 6-month home-based exercise program. The tele group completed 138 min/week versus 58 min/week in control (*p* < 0.02) and also achieved a 5.5% vs. 4.9% increase in peak VO_2_, without significant improvements in HbA1c or quality of life. In another study, Yuan et al. [90] assessed the efficacy of a mobile-based tele-rehabilitation program in patients with T2DM and HF with preserved ejection fraction and concluded that the tele-rehab program was non-inferior to the center CR in terms of functional outcomes (6 min walk distance) as well as frailty status or quality of life.

The implementation of digital health interventions in CR faces several real-world challenges that extend beyond their proven efficacy in controlled trials [91].

At the patient level, barriers such as limited digital literacy, unequal access to devices or reliable internet, and difficulties with sustained engagement can hinder uptake, particularly among older or socioeconomically disadvantaged groups. From the healthcare system perspective, challenges include integrating large volumes of remote monitoring data into existing clinical workflows, ensuring interoperability with electronic health records, and providing adequate training for providers to interpret and act on digital outputs [92,93,94]. Technological limitations, including variable device accuracy, connectivity issues, and frequent software updates, may also disrupt consistent use. Furthermore, broader concerns about data privacy, regulatory compliance, reimbursement models, and the risk of widening health disparities underscore the need for careful planning. Addressing these barriers is essential to unlock the full potential of wearables, telemedicine, and other digital tools in improving cardiac rehabilitation outcomes in routine practice [95,96,97].

For patients diagnosed with type 2 diabetes, the challenges associated with the integration of digital health interventions within cardiac rehabilitation programs are particularly pronounced. A significant proportion of these individuals are of advanced age, and they frequently exhibit a higher prevalence of comorbidities, including neuropathy, retinopathy, and cognitive impairment. These conditions can restrict their capacity to effectively utilize wearable technology or mobile applications [98,99].

Glycemic variability introduces a layer of complexity, as the integration of glucose monitoring data with cardiac rehabilitation platforms necessitates sophisticated interoperability, which is not universally available in clinical practice. Sustained engagement may also be more arduous due to treatment burden, as T2DM patients often face polypharmacy, dietary restrictions, and frequent medical visits. Furthermore, socioeconomic disparities are particularly salient in this demographic, where constrained access to digital tools or reliable internet may exacerbate existing inequalities in healthcare. Ensuring data security and privacy is imperative, given the volume and sensitivity of the combined cardiometabolic and glucose-related information that is collected [99,100,101,102].

Addressing these diabetes-specific barriers is essential to ensure that digital health innovations in cardiac rehabilitation are inclusive, effective, and equitable for this high-risk group of patients.

## 6. Conclusions

Patients with T2DM at very high risk require multidisciplinary and tailored management to improve long-term prognosis. Cardiovascular prevention and CR are two pillars to achieve this goal. The CR program integrates the benefits of personalized exercise training with lifestyle measures, cardiovascular risk factor management, psychosocial support, and optimization of drug treatment. Exercise helps to improve cardiorespiratory fitness, glycemic control, modifiable risk factors and contributes to reducing the risk of long-term morbidity and mortality. CR programs for these patients aim to assess the macrovascular, microvascular, and psychosocial status and complications related to diabetic foot care. Last but not least, an important aspect is glycemic control before, during, and after exercise to prevent acute complications such as hypo- or hyperglycemia. Continuous monitoring, individualized goals, and increased quality of life are aspects that contribute to increasing the compliance of diabetic patients and thus reduce the risk of acute cardiovascular events. Future research should address gaps concerning optimal exercise prescription and long-term adherence strategies for this high-risk patient group.

## Figures and Tables

**Table 1:** The main recommendations on cardiovascular prevention in type 2 diabetes patients.

Area	Recommendation	Impact on CVD Outcomes	Impact on DM Outcomes	Practical Examples for Implementation
Fat intake	Saturated fats < 10% of energy intake, replaced with poly-/monounsaturated fats. Limit trans fats.	Reduces LDL-C	Fat mass reduction improves insulin sensitivity	Replace butter with olive oil; consume 30 g nuts/day
Protein intake	Limit red meat to 350–500 g/week. Include fatty fish (1–2 servings/week). Promotion of plant-based patterns.	Decreases CVD risk	May potentially improve glycemic control	Eat salmon or mackerel 1–2 x/week; limit beef/pork intake.
Carbohydrate intake	Focus on low-glycemic index foods. Fiber intake 30–45 g/day. Limit sugar intake.	Lowers blood pressure, improves lipid profile, and reduces CVD risk	Stabilizes blood glucose levels and reduces postprandial glycemic spikes	Use whole-grain bread/pasta, add legumes to meals.
Salt intake	Limit sodium intake to <5 g/day	Reduces blood pressure, lowering hypertension-related CVD risk	Minimizes fluid retention	Avoid processed foods; replace salt with spices/herbs.
Alcohol	Limit to <100 g/week (class I, level A [5])	Prevents blood pressure elevation and BMI increase	Reduces risk of glycemia fluctuations	Introduce alcohol-free days; educate about standard drink units.
Dietary pattern	Mediterranean or DASH diet (class I, level A [5])	Associated with reduced ASCVD risk and improved lipid profile	Supports long-term glycemic control and weight loss	Provide weekly menu examples and shopping lists.
Physical activity	150 min/week moderate aerobic or 75 min vigorous (class I, level A [5]); RT 2–3 sessions/week	Lowers blood pressure and improves lipid profile	Enhances insulin sensitivity and aids in weight loss	Walking/cycling 30 min/day; use mobile apps for tracking.
Smoking	Complete cessation of smoking (class I, level A [5])	Reduces ASCVD risk significantly	Improves overall metabolic function	Behavioral counseling; pharmacological support if needed.
Psychosocial factors	Screening for anxiety, depression, stress	Reduces ASCVD risk via stress management	Improves adherence to self-care	Regular questionnaires; psychotherapy; mindfulness techniques.

ASCVD, atherosclerotic cardiovascular disease; BMI, body mass index; CVD, cardiovascular disease; DASH, Dietary Approaches to Stop Hypertension; DM, diabetes mellitus; LDL-C, low-density lipoprotein-cholesterol.

**Table 2:** Exercise training in patients with T2DM.

Training Type	Recommendation	Details
Aerobic exercise	At least 150 min/week of moderate-intensity OR 75 min/week of vigorous-intensity activity	Spread over ≥ 3 days/week, with no more than 2 consecutive days without exercise; examples: brisk walking, cycling, swimming
Resistance training	2–3 sessions/week involving all major muscle groups	Intensity: ~70–85% of 1-RM (8–10 repetitions, 2–3 sets);
Flexibility training	At least 2–3 times/week	Static stretching for major muscle groups; hold 10–30 s per stretch
Balance training	Recommended especially for older adults or those with neuropathy	Activities such as tai chi, yoga, balance boards, or targeted physiotherapy
Sedentary behavior	Avoid prolonged sitting; break up sedentary time every 30 min with light activity	Light walking, standing, or stretching breaks
Precautions	Monitor blood glucose before/after exercise, especially if on insulin or sulfonylureas	Avoid exercise if uncontrolled hyperglycemia (>250 mg/dL with ketones) or severe hypoglycemia; ensure proper footwear to prevent foot injury
Progression	Start gradually, especially if sedentary, and increase duration/intensity progressively	Tailor to individual comorbidities, fitness level, and complications (e.g., neuropathy, retinopathy, CVD)

1-RM, one-repetition maximum; CVD, cardiovascular disease; T2DM, type 2 diabetes mellitus.

**Table 3:** Referral to cardiac rehabilitation: barriers and possible solutions for patients with T2DM.

Barrier Domain	Potential Solutions
Low referral & enrollment	Automatic/standardized referral systems; education campaigns.
Lower participation in T2DM	Tailored CR programs for diabetes (glucose monitoring, weight management); gender-sensitive interventions.
Capacity & geographic access	Expand community- or home-based CR; hybrid/digital delivery;
Workforce shortages & training gaps	Task-shifting (nurses, physiotherapists); specialized training in diabetes-focused CR; investment in CR infrastructure.
Patient costs & reimbursement	Ensure insurance reimbursement; government subsidies; flexible scheduling; financial counseling.
Transportation & logistics	Home- or tele-rehabilitation; transport support services; flexible program hours.
Comorbidities & complexity	Multidisciplinary care (cardiology, diabetology, nephrology); individualized exercise prescriptions; closer monitoring.
Fragmented diabetes integration	Integrate SMBG/CGM data; diabetes-specific education; dietitian and diabetes educator involvement.
Provider awareness & endorsement	Provider training; audit and feedback; embed CR promotion in discharge protocols.
System-level barriers	Health IT integration; leadership engagement; funding for hybrid CR models.
Guideline–practice gap	Stronger implementation frameworks; policy mandates; quality indicators tied to referral/participation.

CR, Cardiac Rehabilitation; CGM, Continuous Glucose Monitoring; SMBG, Self-Monitoring of Blood Glucose; T2DM, Type 2 Diabetes Mellitus.

**Table 4:** Structured clinical management checklist in patients with high-risk T2DM.

Domain	Key Components
Risk Stratification	- Assess HbA1c, eGFR, albuminuria, lipids, BP, BMI, waist circumference - Evaluate ASCVD, HF, CKD, neuropathy, retinopathy - Functional/frailty and psychosocial screening
Glycemic Targets	- Individualized HbA1c: <7% if young/low hypoglycemia risk, 7–8% if frail or high risk - Avoid overtreatment and hypoglycemia
Lifestyle Management	- Medical nutrition therapy (renal- or CV-appropriate) - Physical activity tailored to abilities - Structured weight management - Smoking cessation, alcohol moderation
Glucose-Lowering Therapy	- SGLT2 inhibitors: CV, renal, HF protection - GLP-1 receptor agonists: CV protection, weight loss - Metformin: continue if tolerated - Insulin/SUs: use cautiously, avoid hypoglycemia
Cardiometabolic Risk Reduction	- BP control: ACEi/ARB in CKD/albuminuria - Lipid management: statins ± ezetimibe/PCSK9i - Antiplatelet therapy: aspirin in established ASCVD - Renal protection: SGLT2i, RAAS blockade
Complications Management	- Annual retinal screening, neuropathy check, urine albumin - Early cardiology/nephrology referral in CVD/CKD/HF - Multidisciplinary management of foot ulcers, vision loss
Patient-Centered Care	- Shared decision making - Education: SMBG/CGM, hypoglycemia prevention - Psychosocial support, address treatment burden - Consider affordability and access
Team-Based Care	- Involve endocrinology, cardiology, nephrology, dietitian, diabetes educator, psychology - Regular multidisciplinary reviews - Telemedicine and digital tools
Monitoring & Follow-up	- HbA1c every 3–6 months - eGFR & albuminuria at least annually (more frequently in patients with CKD) - Lipids, BP, weight routinely - Adjust therapy based on outcomes and safety

ASCVD, atherosclerotic cardiovascular disease; BP, blood pressure; CKD, chronic kidney disease; CV, cardiovascular; CVD, cardiovascular disease; eGFR, estimated glomerular filtration rate; GLP-1 RA, glucagon-like peptide-1 receptor agonist; HbA1c, glycated hemoglobin; HF, heart failure; MI, myocardial infarction; PCSK9i, proprotein convertase subtilisin/kexin type 9 inhibitor; RAAS, renin–angiotensin–aldosterone system; SMBG/CGM, self-monitoring of blood glucose/continuous glucose monitoring; SGLT2i, sodium–glucose cotransporter-2 inhibitor; SU, sulfonylurea.

## References

[1] Mattina A., Argano C., Brunori G., Lupo U., Raspanti M., Lo Monaco M., Bocchio R.M., Natoli G., Giusti M.A., Corrao S. (2022). Clinical Complexity and Diabetes: A Multidimensional Approach for the Management of Cardiorenal Metabolic Syndrome. Nutr. Metab. Cardiovasc. Dis..

[2] Ozkan B., Ndumele C.E. (2023). Addressing Cardiovascular Risk in Diabetes: It’s More Than the Sugar. Circulation.

[3] Gyldenkerne C., Kahlert J., Thrane P.G., Olesen K.K.W., Mortensen M.B., Sørensen H.T., Thomsen R.W., Maeng M. (2024). 2-Fold More Cardiovascular Disease Events Decades Before Type 2 Diabetes Diagnosis. J. Am. Coll. Cardiol..

[4] Dal Canto E., Ceriello A., Rydén L., Ferrini M., Hansen T.B., Schnell O., Standl E., Beulens J.W. (2019). Diabetes as a Cardiovascular Risk Factor: An Overview of Global Trends of Macro and Micro Vascular Complications. Eur. J. Prev. Cardiol..

[5] Marx N., Federici M., Schütt K., Müller-Wieland D., Ajjan R.A., Antunes M.J., Christodorescu R.M., Crawford C., Di Angelantonio E., Eliasson B. (2023). 2023 ESC Guidelines for the Management of Cardiovascular Disease in Patients with Diabetes. Eur. Heart J..

[6] Pennells L., Kaptoge S., Østergaard H.B., Read S.H., Carinci F., Franch-Nadal J., Petitjean C., Taylor O., Hageman S.H.J., SCORE2-Diabetes Working Group and the ESC Cardiovascular Risk Collaboration (2023). SCORE2-Diabetes: 10-Year Cardiovascular Risk Estimation in Type 2 Diabetes in Europe. Eur. Heart J..

[7] Critchley J.A., Capewell S. (2003). Mortality Risk Reduction Associated with Smoking Cessation in Patients with Coronary Heart Disease: A Systematic Review. JAMA.

[8] Mozaffarian D., Micha R., Wallace S. (2010). Effects on Coronary Heart Disease of Increasing Polyunsaturated Fat in Place of Saturated Fat: A Systematic Review and Meta-Analysis of Randomized Controlled Trials. PLoS Med..

[9] Visseren F.L.J., Mach F., Smulders Y.M., Carballo D., Koskinas K.C., Bäck M., Benetos A., Biffi A., Boavida J.-M., Capodanno D. (2021). 2021 ESC Guidelines on Cardiovascular Disease Prevention in Clinical Practice. Eur. Heart J..

[10] Mozaffarian D., Katan M.B., Ascherio A., Stampfer M.J., Willett W.C. (2006). Trans Fatty Acids and Cardiovascular Disease. N. Engl. J. Med..

[11] Cook N.R., Appel L.J., Whelton P.K. (2014). Lower Levels of Sodium Intake and Reduced Cardiovascular Risk. Circulation.

[12] Kahleova H., Levin S., Barnard N. (2017). Cardio-Metabolic Benefits of Plant-Based Diets. Nutrients.

[13] Qian F., Liu G., Hu F.B., Bhupathiraju S.N., Sun Q. (2019). Association Between Plant-Based Dietary Patterns and Risk of Type 2 Diabetes: A Systematic Review and Meta-Analysis. JAMA Intern. Med..

[14] McMacken M., Shah S. (2017). A Plant-Based Diet for the Prevention and Treatment of Type 2 Diabetes. J. Geriatr. Cardiol. JGC.

[15] Dinu M., Abbate R., Gensini G.F., Casini A., Sofi F. (2017). Vegetarian, Vegan Diets and Multiple Health Outcomes: A Systematic Review with Meta-Analysis of Observational Studies. Crit. Rev. Food Sci. Nutr..

[16] Satija A., Bhupathiraju S.N., Spiegelman D., Chiuve S.E., Manson J.E., Willett W., Rexrode K.M., Rimm E.B., Hu F.B. (2017). Healthful and Unhealthful Plant-Based Diets and the Risk of Coronary Heart Disease in U.S. Adults. J. Am. Coll. Cardiol..

[17] Gan Z.H., Cheong H.C., Tu Y.-K., Kuo P.-H. (2021). Association Between Plant-Based Dietary Patterns and Risk of Cardiovascular Disease: A Systematic Review and Meta-Analysis of Prospective Cohort Studies. Nutrients.

[18] Yokoyama Y., Barnard N.D., Levin S.M., Watanabe M. (2014). Vegetarian Diets and Glycemic Control in Diabetes: A Systematic Review and Meta-Analysis. Cardiovasc. Diagn. Ther..

[19] Reynolds A.N., Akerman A.P., Mann J. (2020). Dietary Fibre and Whole Grains in Diabetes Management: Systematic Review and Meta-Analyses. PLoS Med..

[20] Threapleton D.E., Greenwood D.C., Evans C.E.L., Cleghorn C.L., Nykjaer C., Woodhead C., Cade J.E., Gale C.P., Burley V.J. (2013). Dietary Fibre Intake and Risk of Cardiovascular Disease: Systematic Review and Meta-Analysis. BMJ.

[21] ElSayed N.A., McCoy R.G., Aleppo G., Balapattabi K., Beverly E.A., Briggs Early K., Bruemmer D., Echouffo-Tcheugui J.B., Ekhlaspour L., American Diabetes Association Professional Practice Committee (2025). 8. Obesity and Weight Management for the Prevention and Treatment of Type 2 Diabetes: Standards of Care in Diabetes–2025. Diabetes Care.

[22] Minzer S., Losno R.A., Casas R. (2020). The Effect of Alcohol on Cardiovascular Risk Factors: Is There New Information?. Nutrients.

[23] Estruch R., Ros E., Salas-Salvadó J., Covas M.-I., Corella D., Arós F., Gómez-Gracia E., Ruiz-Gutiérrez V., Fiol M., Lapetra J. (2018). Primary Prevention of Cardiovascular Disease with a Mediterranean Diet Supplemented with Extra-Virgin Olive Oil or Nuts. N. Engl. J. Med..

[24] Sacks F.M., Svetkey L.P., Vollmer W.M., Appel L.J., Bray G.A., Harsha D., Obarzanek E., Conlin P.R., Miller E.R., Simons-Morton D.G. (2001). Effects on Blood Pressure of Reduced Dietary Sodium and the Dietary Approaches to Stop Hypertension (DASH) Diet. DASH-Sodium Collaborative Research Group. N. Engl. J. Med..

[25] Bakker E.A., Lee D., Hopman M.T.E., Oymans E.J., Watson P.M., Thompson P.D., Thijssen D.H.J., Eijsvogels T.M.H. (2021). Dose–Response Association Between Moderate to Vigorous Physical Activity and Incident Morbidity and Mortality for Individuals with a Different Cardiovascular Health Status: A Cohort Study Among 142,493 Adults from the Netherlands. PLoS Med..

[26] McEvoy J.W., McCarthy C.P., Bruno R.M., Brouwers S., Canavan M.D., Ceconi C., Christodorescu R.M., Daskalopoulou S.S., Ferro C.J., Gerdts E. (2024). 2024 ESC Guidelines for the Management of Elevated Blood Pressure and Hypertension. Eur. Heart J..

[27] Mach F., Baigent C., Catapano A.L., Koskinas K.C., Casula M., Badimon L., Chapman M.J., De Backer G.G., Delgado V., Ference B.A. (2020). 2019 ESC/EAS Guidelines for the Management of Dyslipidaemias: Lipid Modification to Reduce Cardiovascular Risk. Eur. Heart J..

[28] McDonagh T.A., Metra M., Adamo M., Gardner R.S., Baumbach A., Böhm M., Burri H., Butler J., Čelutkienė J., Chioncel O. (2021). 2021 ESC Guidelines for the Diagnosis and Treatment of Acute and Chronic Heart Failure. Eur. Heart J..

[29] Chan J.S.K., Perone F., Bayatpoor Y., Tse G., Harky A. (2023). Emerging Sodium-Glucose Cotransporter-2 Inhibitor Therapies for Managing Heart Failure in Patients with Chronic Kidney Disease. Expert Opin. Pharmacother..

[30] Armillotta M., Angeli F., Paolisso P., Belmonte M., Raschi E., Di Dalmazi G., Amicone S., Canton L., Fedele D., Suma N. (2025). Cardiovascular Therapeutic Targets of Sodium-Glucose Co-Transporter 2 (SGLT2) Inhibitors Beyond Heart Failure. Pharmacol. Ther..

[31] Mariani M.V., Manzi G., Pierucci N., Laviola D., Piro A., D’Amato A., Filomena D., Matteucci A., Severino P., Miraldi F. (2024). SGLT2i Effect on Atrial Fibrillation: A Network Meta-Analysis of Randomized Controlled Trials. J. Cardiovasc. Electrophysiol..

[32] Chen X., Zhang X., Xiang X., Fang X., Feng S. (2024). Effects of Glucagon-like Peptide-1 Receptor Agonists on Cardiovascular Outcomes in High-Risk Type 2 Diabetes: A Systematic Review and Meta-Analysis of Randomized Controlled Trials. Diabetol. Metab. Syndr..

[33] Mullur N., Morissette A., Morrow N.M., Mulvihill E.E. (2024). GLP-1 Receptor Agonist-Based Therapies and Cardiovascular Risk: A Review of Mechanisms. J. Endocrinol..

[34] Odigwe C., Mulyala R., Malik H., Ruiz B., Riad M., Sayiadeh M.A., Honganur S., Parks A., Rahman M.U., Lakkis N. (2025). Emerging Role of GLP-1 Agonists in Cardio-Metabolic Therapy—Focus on Semaglutide. Am. Heart J. Plus Cardiol. Res. Pract..

[35] Vaccarino V., Badimon L., Bremner J.D., Cenko E., Cubedo J., Dorobantu M., Duncker D.J., Koller A., Manfrini O., Milicic D. (2020). Depression and Coronary Heart Disease: 2018 Position Paper of the ESC Working Group on Coronary Pathophysiology and Microcirculation. Eur. Heart J..

[36] Bueno H., Deaton C., Farrero M., Forsyth F., Braunschweig F., Buccheri S., Dragan S., Gevaert S., Held C., Kurpas D. (2025). 2025 ESC Clinical Consensus Statement on Mental Health and Cardiovascular Disease: Developed Under the Auspices of the ESC Clinical Practice Guidelines Committee. Eur. Heart J..

[37] Pillinger T., McCutcheon R.A., Vano L., Mizuno Y., Arumuham A., Hindley G., Beck K., Natesan S., Efthimiou O., Cipriani A. (2020). Comparative Effects of 18 Antipsychotics on Metabolic Function in Patients with Schizophrenia, Predictors of Metabolic Dysregulation, and Association with Psychopathology: A Systematic Review and Network Meta-Analysis. Lancet Psychiatry.

[38] Simon V., van Winkel R., De Hert M. (2009). Are Weight Gain and Metabolic Side Effects of Atypical Antipsychotics Dose Dependent? A Literature Review. J. Clin. Psychiatry.

[39] Piras M., Chahma J., Ranjbar S., Laaboub N., Grosu C., Plessen K.J., von Gunten A., Conus P., Eap C.B. (2023). Is Clozapine-Induced Weight Gain Dose-Dependent? Results from a Prospective Cohort Study. Schizophr. Bull..

[40] Singh M., Stewart R., White H. (2014). Importance of Frailty in Patients with Cardiovascular Disease. Eur. Heart J..

[41] Newby D.E., Mannucci P.M., Tell G.S., Baccarelli A.A., Brook R.D., Donaldson K., Forastiere F., Franchini M., Franco O.H., Graham I. (2015). Expert Position Paper on Air Pollution and Cardiovascular Disease. Eur. Heart J..

[42] Kemps H., Kränkel N., Dörr M., Moholdt T., Wilhelm M., Paneni F., Serratosa L., Ekker Solberg E., Hansen D., Halle M. (2019). Exercise Training for Patients with Type 2 Diabetes and Cardiovascular Disease: What to Pursue and How to Do It. A Position Paper of the European Association of Preventive Cardiology (EAPC). Eur. J. Prev. Cardiol..

[43] Ambrosetti M., Fattirolli F., Maranta F., Ruzzolini M., Rizzo M., Mureddu G.F., Griffo R., Venturini E., Giallauria F., Orso F. (2023). La Gestione Del Paziente Con Diabete Di Tipo 2 in Cardiologia Preventiva e Riabilitativa. Expert Opinion Della Italian Alliance for Cardiovascular Rehabilitation and Prevention (ITACARE-P). G. Ital. Cardiol..

[44] Ambrosetti M., Abreu A., Corrà U., Davos C.H., Hansen D., Frederix I., Iliou M.C., Pedretti R.F.E., Schmid J.-P., Vigorito C. (2021). Secondary Prevention through Comprehensive Cardiovascular Rehabilitation: From Knowledge to Implementation. 2020 Update. A Position Paper from the Secondary Prevention and Rehabilitation Section of the European Association of Preventive Cardiology. Eur. J. Prev. Cardiol..

[45] Hansen D., Kraenkel N., Kemps H., Wilhelm M., Abreu A., Pfeiffer A.F., Jordão A., Cornelissen V., Völler H. (2019). Management of Patients with Type 2 Diabetes in Cardiovascular Rehabilitation. Eur. J. Prev. Cardiol..

[46] Blioumpa C., Karanasiou E., Antoniou V., Batalik L., Kalatzis K., Lanaras L., Pepera G. (2023). Efficacy of Supervised Home-Based, Real Time, Videoconferencing Telerehabilitation in Patients with Type 2 Diabetes: A Single-Blind Randomized Controlled Trial. Eur. J. Phys. Rehabil. Med..

[47] Pepera G., Karanasiou E., Blioumpa C., Antoniou V., Kalatzis K., Lanaras L., Batalik L. (2023). Tele-Assessment of Functional Capacity through the Six-Minute Walk Test in Patients with Diabetes Mellitus Type 2: Validity and Reliability of Repeated Measurements. Sensors.

[48] Buckley J.P., Riddell M., Mellor D., Bracken R.M., Ross M.-K., LaGerche A., Poirier P. (2021). Acute Glycaemic Management before, During and after Exercise for Cardiac Rehabilitation Participants with Diabetes Mellitus: A Joint Statement of the British and Canadian Associations of Cardiovascular Prevention and Rehabilitation, the International Council for Cardiovascular Prevention and Rehabilitation and the British Association of Sport and Exercise Sciences. Br. J. Sports Med..

[49] Mendes R., Sousa N., Almeida A., Subtil P., Guedes-Marques F., Reis V.M., Themudo-Barata J.L. (2016). Exercise Prescription for Patients with Type 2 Diabetes—A Synthesis of International Recommendations: Narrative Review. Br. J. Sports Med..

[50] Hordern M.D., Dunstan D.W., Prins J.B., Baker M.K., Singh M.A.F., Coombes J.S. (2012). Exercise Prescription for Patients with Type 2 Diabetes and Pre-Diabetes: A Position Statement from Exercise and Sport Science Australia. J. Sci. Med. Sport.

[51] Perone F., Pingitore A., Conte E., Halasz G., Ambrosetti M., Peruzzi M., Cavarretta E. (2023). Obesity and Cardiovascular Risk: Systematic Intervention Is the Key for Prevention. Healthcare.

[52] Sigal R.J., Kenny G.P., Wasserman D.H., Castaneda-Sceppa C., White R.D. (2006). Physical Activity/Exercise and Type 2 Diabetes: A Consensus Statement from the American Diabetes Association. Diabetes Care.

[53] Su W., Tao M., Ma L., Tang K., Xiong F., Dai X., Qin Y. (2023). Dose-Response Relationships of Resistance Training in Type 2 Diabetes Mellitus: A Meta-Analysis of Randomized Controlled Trials. Front. Endocrinol..

[54] Paneni F., Beckman J.A., Creager M.A., Cosentino F. (2013). Diabetes and Vascular Disease: Pathophysiology, Clinical Consequences, and Medical Therapy: Part I. Eur. Heart J..

[55] Boulé N.G., Kenny G.P., Haddad E., Wells G.A., Sigal R.J. (2003). Meta-Analysis of the Effect of Structured Exercise Training on Cardiorespiratory Fitness in Type 2 Diabetes Mellitus. Diabetologia.

[56] Church T.S., Blair S.N., Cocreham S., Johannsen N., Johnson W., Kramer K., Mikus C.R., Myers V., Nauta M., Rodarte R.Q. (2010). Effects of Aerobic and Resistance Training on Hemoglobin A_1c_ Levels in Patients with Type 2 Diabetes: A Randomized Controlled Trial. JAMA.

[57] Hansen D., Niebauer J., Cornelissen V., Barna O., Neunhäuserer D., Stettler C., Tonoli C., Greco E., Fagard R., Coninx K. (2018). Exercise Prescription in Patients with Different Combinations of Cardiovascular Disease Risk Factors: A Consensus Statement from the EXPERT Working Group. Sports Med..

[58] Jansson A.K., Chan L.X., Lubans D.R., Duncan M.J., Plotnikoff R.C. (2022). Effect of Resistance Training on HbA1c in Adults with Type 2 Diabetes Mellitus and the Moderating Effect of Changes in Muscular Strength: A Systematic Review and Meta-Analysis. BMJ Open Diabetes Res. Care.

[59] Pelliccia A., Sharma S., Gati S., Bäck M., Börjesson M., Caselli S., Collet J.-P., Corrado D., Drezner J.A., Halle M. (2021). 2020 ESC Guidelines on Sports Cardiology and Exercise in Patients with Cardiovascular Disease. Eur. Heart J..

[60] Chudyk A., Petrella R.J. (2011). Effects of Exercise on Cardiovascular Risk Factors in Type 2 Diabetes. Diabetes Care.

[61] Ruas J.L., White J.P., Rao R.R., Kleiner S., Brannan K.T., Harrison B.C., Greene N.P., Wu J., Estall J.L., Irving B.A. (2012). A PGC-1α Isoform Induced by Resistance Training Regulates Skeletal Muscle Hypertrophy. Cell.

[62] Figueira F.R., Umpierre D., Cureau F.V., Zucatti A.T.N., Dalzochio M.B., Leitão C.B., Schaan B.D. (2014). Association Between Physical Activity Advice Only or Structured Exercise Training with Blood Pressure Levels in Patients with Type 2 Diabetes: A Systematic Review and Meta-Analysis. Sports Med..

[63] Hansen D., Abreu A., Ambrosetti M., Cornelissen V., Gevaert A., Kemps H., Laukkanen J.A., Pedretti R., Simonenko M., Wilhelm M. (2022). Exercise Intensity Assessment and Prescription in Cardiovascular Rehabilitation and Beyond: Why and How: A Position Statement from the Secondary Prevention and Rehabilitation Section of the European Association of Preventive Cardiology. Eur. J. Prev. Cardiol..

[64] D’Ascenzi F., Cavigli L., Pagliaro A., Focardi M., Valente S., Cameli M., Mandoli G.E., Mueller S., Dendale P., Piepoli M. (2022). Clinician Approach to Cardiopulmonary Exercise Testing for Exercise Prescription in Patients at Risk of and with Cardiovascular Disease. Br. J. Sports Med..

[65] Li H., Tao L., Huang Y., Li Z., Zhao J. (2022). Inspiratory Muscle Training in Patients with Heart Failure: A Systematic Review and Meta-Analysis. Front. Cardiovasc. Med..

[66] Sadek Z., Salami A., Joumaa W.H., Awada C., Ahmaidi S., Ramadan W. (2018). Best Mode of Inspiratory Muscle Training in Heart Failure Patients: A Systematic Review and Meta-Analysis. Eur. J. Prev. Cardiol..

[67] Moreno A.M., Toledo-Arruda A.C., Lima J.S., Duarte C.S., Villacorta H., Nóbrega A.C.L. (2017). Inspiratory Muscle Training Improves Intercostal and Forearm Muscle Oxygenation in Patients with Chronic Heart Failure: Evidence of the Origin of the Respiratory Metaboreflex. J. Card. Fail..

[68] Mazzolai L., Teixido-Tura G., Lanzi S., Boc V., Bossone E., Brodmann M., Bura-Rivière A., De Backer J., Deglise S., Della Corte A. (2024). 2024 ESC Guidelines for the Management of Peripheral Arterial and Aortic Diseases. Eur. Heart J..

[69] Parmenter B.J., Mavros Y., Ritti Dias R., King S., Fiatarone Singh M. (2020). Resistance Training as a Treatment for Older Persons with Peripheral Artery Disease: A Systematic Review and Meta-Analysis. Br. J. Sports Med..

[70] Blears E.E., Elias J.K., Tapking C., Porter C., Rontoyanni V.G. (2021). Supervised Resistance Training on Functional Capacity, Muscle Strength and Vascular Function in Peripheral Artery Disease: An Updated Systematic Review and Meta-Analysis. J. Clin. Med..

[71] Pearson S.J., Sindall P., Caldow E., Taberner P. (2023). The Effect of Resistance Training on Functional Capacity in Middle-Aged to Elderly Individuals with Peripheral Artery Disease: A Meta-Analysis. Int. Angiol..

[72] Abdollahpour Alni M., Nikookheslat S.D. (2022). The Effect of 12 Weeks Aerobic, Resistance and Combined Trainings on Peripheral Vascular Disease in Type 2 Diabetes with Peripheral Neuropathy in Men. Obes. Med..

[73] Orlando G., Balducci S., Boulton A.J.M., Degens H., Reeves N.D. (2022). Neuromuscular Dysfunction and Exercise Training in People with Diabetic Peripheral Neuropathy: A Narrative Review. Diabetes Res. Clin. Pract..

[74] Maestroni L., Read P., Bishop C., Papadopoulos K., Suchomel T.J., Comfort P., Turner A. (2020). The Benefits of Strength Training on Musculoskeletal System Health: Practical Applications for Interdisciplinary Care. Sports Med..

[75] Zanuso S., Sacchetti M., Sundberg C.J., Orlando G., Benvenuti P., Balducci S. (2017). Exercise in Type 2 Diabetes: Genetic, Metabolic and Neuromuscular Adaptations. A Review of the Evidence. Br. J. Sports Med..

[76] Handsaker J.C., Brown S.J., Bowling F.L., Maganaris C.N., Boulton A.J.M., Reeves N.D. (2016). Resistance Exercise Training Increases Lower Limb Speed of Strength Generation During Stair Ascent and Descent in People with Diabetic Peripheral Neuropathy. Diabet. Med..

[77] Thukral N., Kaur J., Malik M. (2021). A Systematic Review and Meta-Analysis on Efficacy of Exercise on Posture and Balance in Patients Suffering from Diabetic Neuropathy. Curr. Diabetes Rev..

[78] Stefanakis M., Batalik L., Papathanasiou J., Dipla L., Antoniou V., Pepera G. (2021). Exercise-Based Cardiac Rehabilitation Programs in the Era of COVID-19: A Critical Review. Rev. Cardiovasc. Med..

[79] Su J.J., Paguio J.T., Wang W., Batalik L. (2025). Designing a Nurse-Led eHealth Cardiac Rehabilitation Program: Insights from Participant Experiences and Qualitative Feedback. Public Health Nurs..

[80] Beleigoli A., Dafny H.A., Pinero De Plaza M.A., Hutchinson C., Marin T., Ramos J.S., Suebkinorn O., Gebremichael L.G., Bulamu N.B., Keech W. (2024). Clinical Effectiveness of Cardiac Rehabilitation and Barriers to Completion in Patients of Low Socioeconomic Status in Rural Areas: A Mixed-Methods Study. Clin. Rehabil..

[81] Fraser M.J., Leslie S.J., Gorely T., Foster E., Walters R. (2022). Barriers and Facilitators to Participating in Cardiac Rehabilitation and Physical Activity: A Cross-Sectional Survey. World J. Cardiol..

[82] Gadager B.B., Tang L.H., Doherty P., Svendsen M.L., Sibilitz K.L., Harrison A., Maribo T. (2024). Are Cardiac Rehabilitation Pathways Influenced by Diabetes: A Cohort Study. Int. J. Cardiol..

[83] Rizza V., Tondi L., Patti A.M., Cecchi D., Lombardi M., Perone F., Ambrosetti M., Rizzo M., Cianflone D., Maranta F. (2024). Diabetic Cardiomyopathy: Pathophysiology, Imaging Assessment and Therapeutical Strategies. Int. J. Cardiol. Cardiovasc. Risk Prev..

[84] Galindo R.J., Trujillo J.M., Low Wang C.C., McCoy R.G. (2023). Advances in the Management of Type 2 Diabetes in Adults. BMJ Med..

[85] Williams D.M., Jones H., Stephens J.W. (2022). Personalized Type 2 Diabetes Management: An Update on Recent Advances and Recommendations. Diabetes Metab. Syndr. Obes. Targets Ther..

[86] Wong N.D., Sattar N. (2023). Cardiovascular Risk in Diabetes Mellitus: Epidemiology, Assessment and Prevention. Nat. Rev. Cardiol..

[87] Bluhm M.L., Hoehing K.N., Nelson R.K., Zuhl M.N. (2022). Effect of Type-2 Diabetes Mellitus on Cardiac Rehabilitation Outcomes: A Meta-Analysis. Arch. Phys. Med. Rehabil..

[88] Wongvibulsin S., Habeos E.E., Huynh P.P., Xun H., Shan R., Porosnicu Rodriguez K.A., Wang J., Gandapur Y.K., Osuji N., Shah L.M. (2021). Digital Health Interventions for Cardiac Rehabilitation: Systematic Literature Review. J. Med. Internet Res..

[89] Marios T., Smart N., Dalton S. (2012). The Effect of Tele-Monitoring on Exercise Training Adherence, Functional Capacity, Quality of Life and Glycemic Control in Patients with Type II Diabetes. J. Sports Sci. Med..

[90] Yuan M., Xu H., Zhao D., Shi D., Su L., Zhu H., Lu S., Wei J. (2024). Tele-Rehabilitation for Type II Diabetics with Heart Failure with Preserved Ejection Fraction. Front. Endocrinol..

[91] Harbi A.S., Soh K.L., Yubbu P.B., Soh K.G. (2024). Digital Health Intervention in Patients Undergoing Cardiac Rehabilitation: Systematic Review and Meta-Analysis. F1000Research.

[92] Tadas S., Coyle D. (2020). Barriers to and Facilitators of Technology in Cardiac Rehabilitation and Self-Management: Systematic Qualitative Grounded Theory Review. J. Med. Internet Res..

[93] Berezin A.E. (2025). Digital Health Interventions for Cardiac Rehabilitation in Stable Coronary Artery Disease. Eur. J. Prev. Cardiol..

[94] Golbus J.R., Lopez-Jimenez F., Barac A., Cornwell W.K., Dunn P., Forman D.E., Martin S.S., Schorr E.N., Supervia M., on Behalf of the Exercise, Cardiac Rehabilitation and Secondary Prevention Committee of the Council on Clinical Cardiology; Council on Lifelong Congenital Heart Disease and Heart Health in the Young; Council on Quality of Care and Outcomes Research; and Council on Cardiovascular and Stroke Nursing (2023). Digital Technologies in Cardiac Rehabilitation: A Science Advisory from the American Heart Association. Circulation.

[95] Braver J., Marwick T.H., Salim A., Hakamuwalekamlage D., Keating C., Yiallourou S.R., Oldenburg B., Carrington M.J. (2025). Effects of a Digitally Enabled Cardiac Rehabilitation Intervention on Risk Factors, Recurrent Hospitalization and Mortality. Eur. Heart J.-Digit. Health.

[96] Falter M., Scherrenberg M., Dendale P. (2021). Digital Health in Cardiac Rehabilitation and Secondary Prevention: A Search for the Ideal Tool. Sensors.

[97] Lunz L., Würth S., Kulnik S.T. (2025). Health Care Professionals’ Use of Digital Technology in the Secondary Prevention of Cardiovascular Disease in Austria: Online Survey Study. JMIR Cardio.

[98] Moschonis G., Siopis G., Jung J., Eweka E., Willems R., Kwasnicka D., Asare B.Y.-A., Kodithuwakku V., Verhaeghe N., Vedanthan R. (2023). Effectiveness, Reach, Uptake, and Feasibility of Digital Health Interventions for Adults with Type 2 Diabetes: A Systematic Review and Meta-Analysis of Randomised Controlled Trials. Lancet Digit. Health.

[99] Mirasghari F., Ayatollahi H., Velayati F., Abasi A. (2024). Challenges of Using Telemedicine for Patients with Diabetes During the COVID-19 Pandemic: A Scoping Review. J. Clin. Transl. Endocrinol..

[100] Mullur R.S., Hsiao J.S., Mueller K. (2022). Telemedicine in Diabetes Care. Am. Fam. Physician.

[101] Alzghaibi H. (2025). Perspectives of People with Diabetes on AI-Integrated Wearable Devices: Perceived Benefits, Barriers, and Opportunities for Self-Management. Front. Med..

[102] Iwaya L.H., Ahmad A., Babar M.A. (2020). Security and Privacy for mHealth and uHealth Systems: A Systematic Mapping Study. IEEE Access.

